# A clinical case concerning an extraordinary maxillary second molar having two separate palatal roots

**DOI:** 10.1002/ccr3.8893

**Published:** 2024-05-06

**Authors:** Aishwarya A. Kottur, Abdul Mujeeb, Niher Tabassum Siddiqua Snigdha, Mohmed Isaqali Karobari

**Affiliations:** ^1^ Department of Conservative Dentistry and Endodontics S.J.M Dental College And Hospital Chitradurga Karnataka India; ^2^ Department of Dental Research, Center for Global health Research, Saveetha Institute of Medical and Technical Sciences Saveetha Medical College and Hospitals, Saveetha University Chennai Tamil Nadu India; ^3^ Department of Restorative Dentistry & Endodontics, Faculty of Dentistry University of Puthisastra Phnom Penh Cambodia

**Keywords:** CBCT, endodontics, maxillary second molar, root canal, two separate palatal canals and roots

## Abstract

**Key Clinical Message:**

Main objective of root canal therapy is to locate all the canals, cleaning and shaping, and obturation to obtain fluid tight seal. Failure to locate all canals can lead to the failure of root canal therapy.

**Abstract:**

Variation of pulp aperture, among teeth with multiple roots, constitutes recurring issue during diagnosing and completing efficient endodontic procedures. Understanding normal anatomy features and associated likely modifications is critical in the effective execution of the dental procedure, since the inability to effectively treat simply one canal may end up into endodontic unsuccessful therapy. The paper covers a procedure whereby the root pattern and canals of the maxillary second molar were modified employing an operative microscope and verified with cone‐beam computed tomography (CBCT). Cone‐beam computed tomography revealed that the maxillary second molar containing two different palatal roots and canals and two distinct buccal roots and canals. This Research paper presents and investigates the morphological difference observed on the maxillary second molar in order to guarantee the effectiveness of root canal treatment examined utilizing imaging techniques like CBCT.

## 1 INTRODUCTION

Intrinsic architecture within a human teeth is characterized by anatomical complexity. A plethora of studies have proven the fanciful complexities of canal system. Numerous variants are clearly articulated and reported, ranging from countless roots to canals, ramifications, lateral canals, fins, and apical deltas. The introduction of innovative equipment to the dental environment, like operating microscopy and cone‐beam computed tomography (CBCT), has resulted in an unprecedented exploration of the system for root canals.

The internal structure of teeth is an important criterion for determining the efficacy of the root canal procedure.^1^ As a result, the outcome of the endodontic procedure is dependent upon the detection and treatment of every single canals.^1^ Because of the uniqueness of every single teeth, there are numerous anatomical nuances imaginable, which increases the difficulties of operations, especially during chemo‐mechanical preparation and sealing canals.^1^

On the contrary, unsatisfactory treatment of root canals because of an incorrect canal sculpting procedure is generally an indication of a lack of understanding of the complicated interior canal architecture.^1^ Three roots are the more prevalent and repeated morphological number of roots recorded in the scientific record in maxillary second molars, which still show that there is inadequate specificity in pinpointing microscopic structures like additional and connecting canals.^1^ These investigations are conducted using multifaceted techniques, like radiography, that are unable to notice minor variations in morphology.^1^

Multiple approaches for examining canal structure are being used, including traditional radiography and, lately, CBCT and micro‐CT.^2^ CBCT serves as a straightforward, safe, and suitable diagnostic approach with a broad spectrum of uses in dentistry, including evaluation of vertical root fractures, treatment of root canal effects, assessing of canal structure and presurgical review.^2^ CBCT provides an excellent level of precision in determining canal architecture.^2^ As a result, it is regarded to be an appropriate and sufficient therapeutic tool to evaluate tooth canal anatomy.^2^

The present paper discusses the effective root canal therapy of permanent maxillary second molar having four canals and four roots.

## 2 CASE HISTORY/EXAMINATION

A 41‐year‐old Indian female patient came to the dentistry division having a significant symptom regarding persistent discomfort in her uppermost posterior teeth area. The individual described pain that had been ongoing for a period of two days. The patient was in great overall wellness and had no previous serious medical conditions.

**FIGURE 1:**
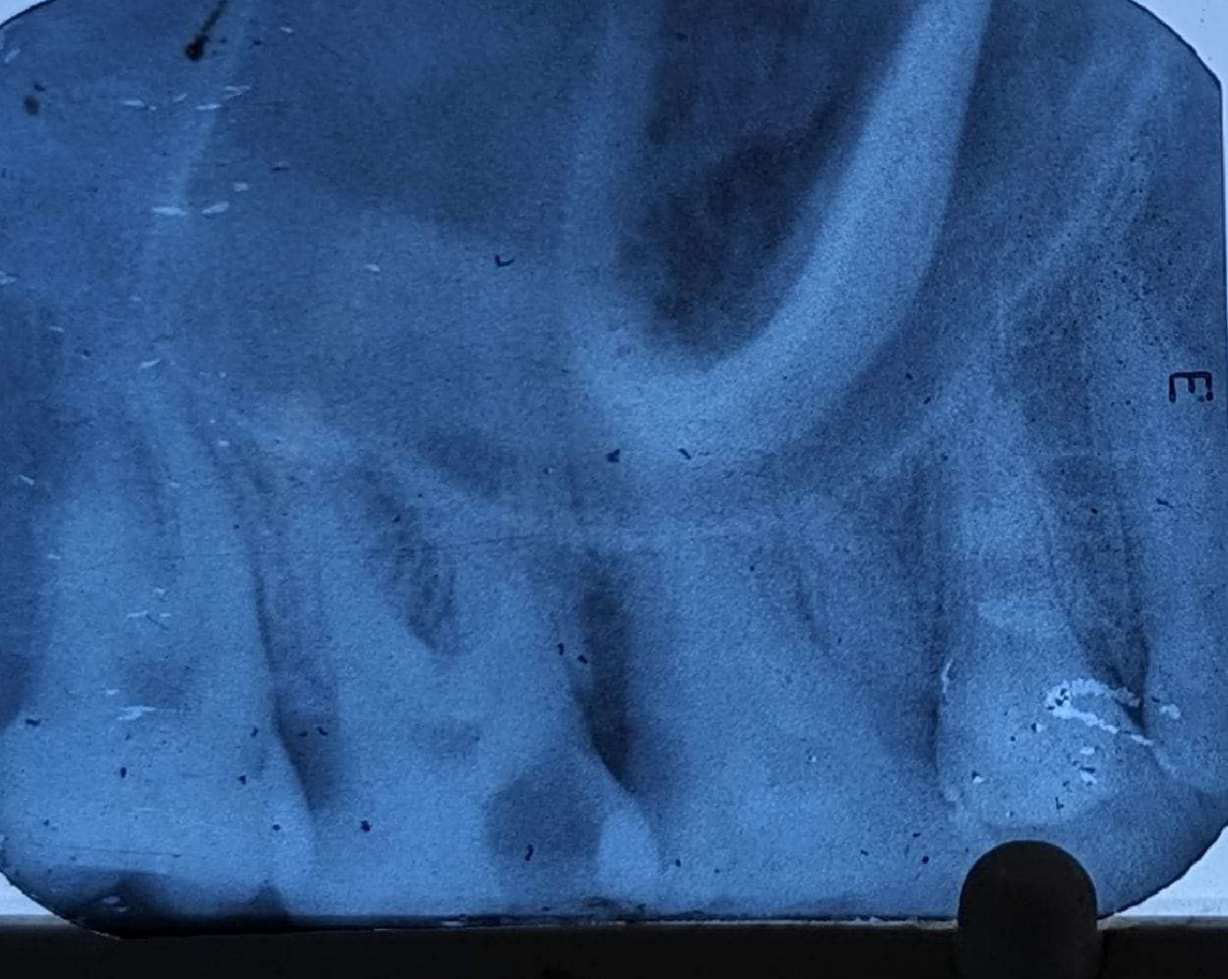
Preoperative radiograph.

A dental examination revealed that tooth 17 showed profound proximal decay along with a cavitated condition. It had hardly extraoral/intraoral swelling, nor was present a drainage channel related with the diagnosed tooth. Connected gingiva appeared and palpated normally. Percussion made the pulp of the tooth sore. Cold test revealed that tooth 17 had a response that was tardy. Tooth 17 had symptomatic irreversible pulpitis and healthy apical structures (Figure 1). Therefore, regarding tooth 17, conventional root canal therapy was intended to be accompanied by complete covering crown.

## 3 METHODS

**FIGURE 2:**
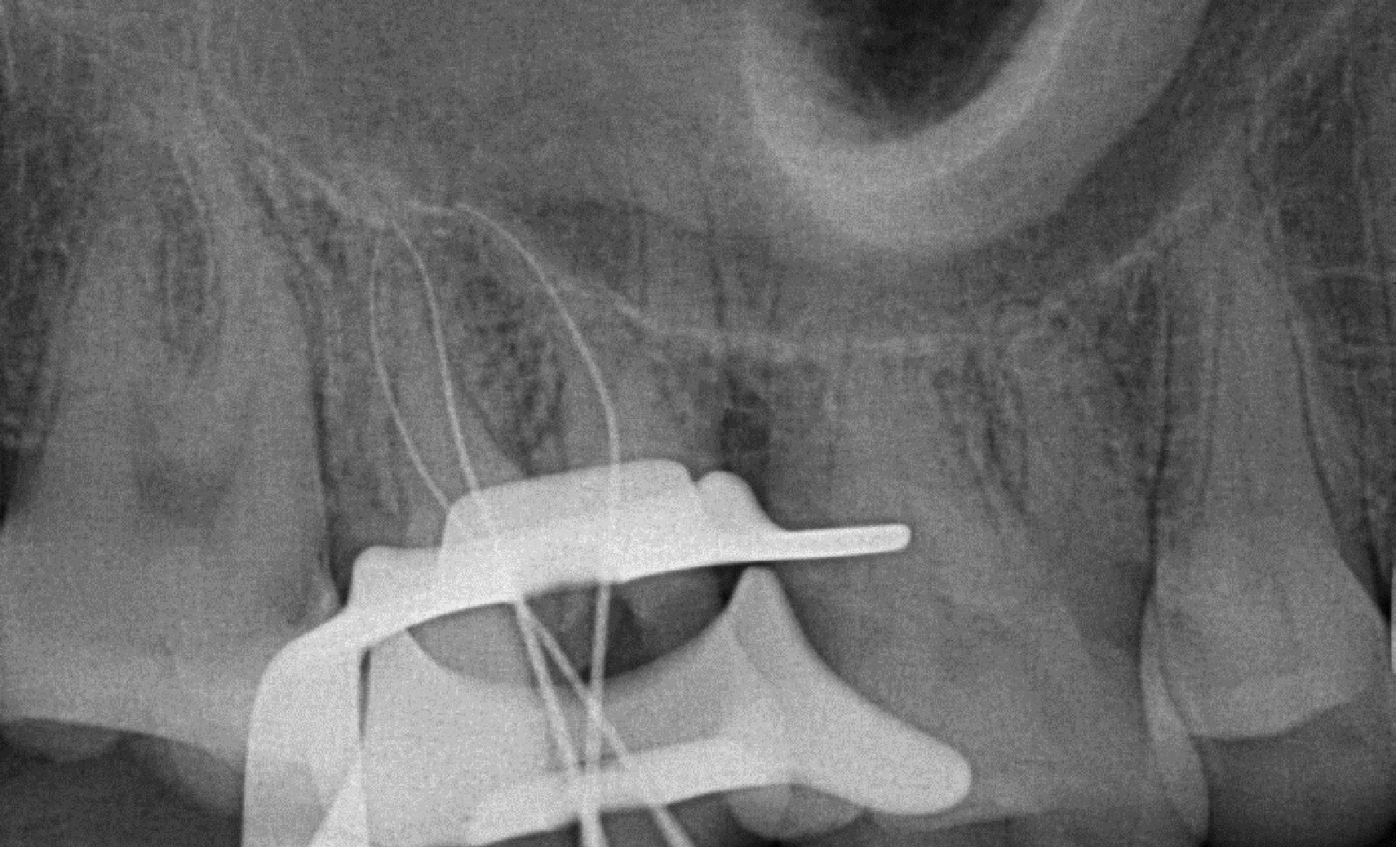
Radiograph image showing the presence of additional root irt tooth 17.

Following receiving the individual's written agreement, local anesthetic was delivered with articaine four percent and adrenaline 1:80,000. The access opening was then created with microscopy using a rubber dam. The existence of four openings in the cavity was established by radiographic evaluation: two in the buccal region and two in the palatal region (Figure 2). The existence of another palatal canal was established using an operational microscopy and a DG‐16 probe.

**FIGURE 3:**
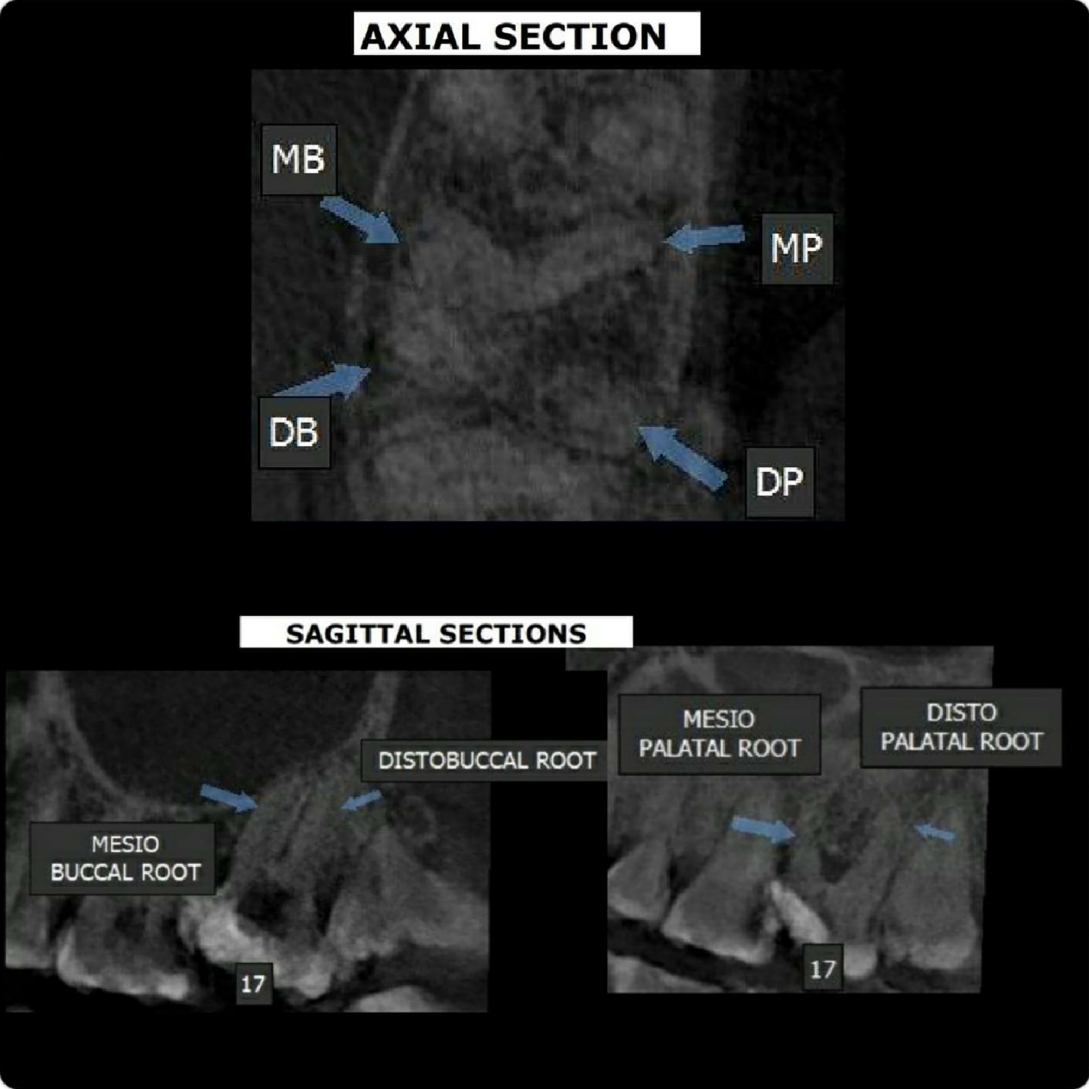
For confirmation: Cone‐beam computed tomography images showing two distinct buccal roots and two distinct palatal roots.

Coronal flaring was carried out following navigating the canals employing no.10 K file (Mani Inc.ltd; Tochigi, Japan). Cavity had been temporarily closed, and the individual was directed to have a CBCT imaging to validate the results. 4‐rooted maxillary second molar comprising a pair of separate palatal roots and canals and a pair of separate buccal roots and canals was found by CBCT (Figure 3).

**FIGURE 4:**
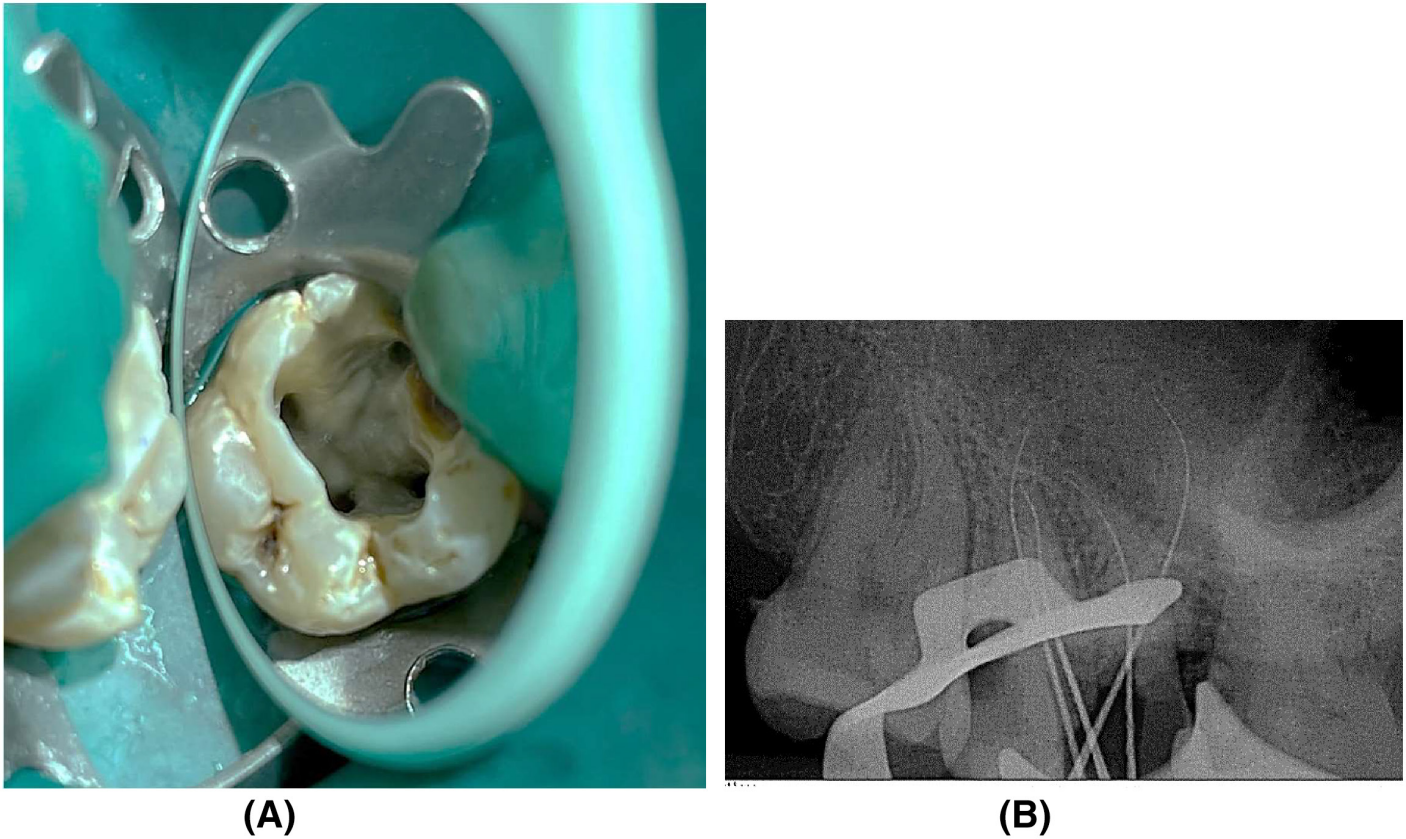
(A) Access opening on maxillary right second molar showing four root canal orifices (B) Radiograph image showing working length determination.

**FIGURE 5:**
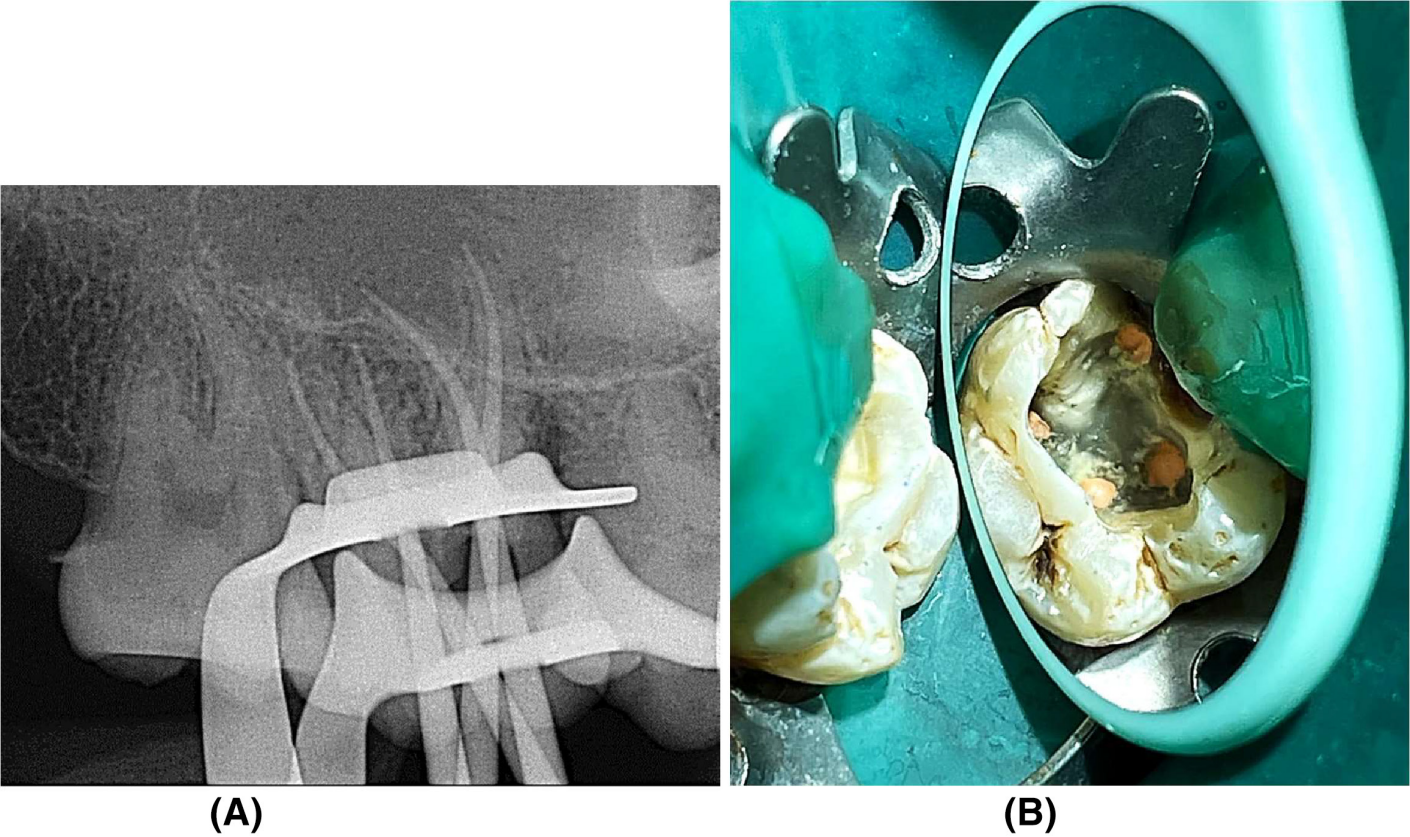
(A) Radiograph image showing master cone (B) Shows complete obturation.

During the subsequent appointment, for easy uninterrupted accessibility to every canals, cavity was altered from being triangular into a square form (Figure 4A) and an electronic apex finder (J Morita Root ZX Mini) was employed to measure working length, which was then verified using radiographs of apical region with multiple angulations (Figure 4B). Dentsply Protaper Gold files were employed to form the canals. MB, DB, MP, and DP orifices were formed to a size F3. Subsequent to acquiring a master cone imaging (Figure 5A), a specific irrigation routine was used: 5.25% sodium hypochlorite (Nice Chemical PVT Ltd, Kochi, India) was stimulated with Endoactivator, then replaced with 17% ethylenediaminetetraacetic acid (Nice Chemical PVT Ltd, Kochi, India) over 1 min in each canal. The very last rinse was normal saline (Infutec Healthcare Limited, Indore, Madhya Pradesh, India). Paper points have been employed to dry canals. Canals were sealed with an AH Plus sealer (Dentsply Sirona) and a heated vertical compaction method (Figure 5B). Composite resin had been employed for restoring the access opening.

## 4 RESULTS

**FIGURE 6:**
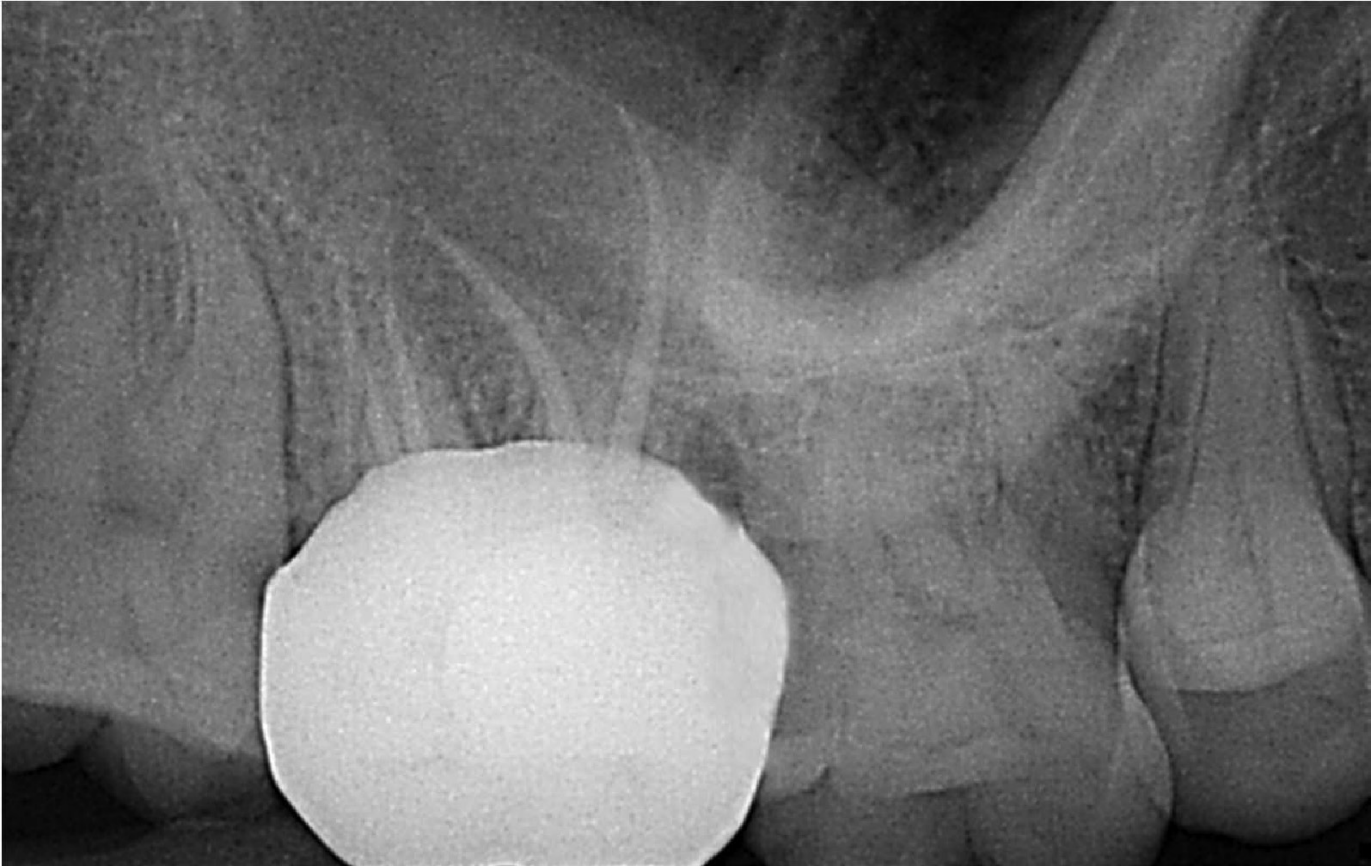
3 months follow‐up.

The Outcome of this case report was to complete the root canal treatment without any chances of failure with the help of CBCT examination. CBCT aided in identifying a separate palatal root which was difficult to find out through traditional radiography. Cleaning and shaping of all the canals and obturation was successful with the help of CBCT. The Main objective of root canal therapy is to locate all the canals, cleaning and shaping and obturation to obtain fluid tight seal. Failure to locate all canals can lead to the failure of root canal therapy, which was prevented in this case report with the help of CBCT. The patient was called for follow‐up after 3 months and the patient was symptom free. (Figure 6).

## 5 DISCUSSION

Four‐rooted maxillary second molars are unusual in study.^3^ Around 1.4% of maxillary molars, Peikoff et al. detected an additional palatal root.^4^ In an in vivo investigation, Hartwell revealed that 9.6% among 176 maxillary second molars contained four canals.^5^ Alani demonstrated the treatment for two palatal roots of contralateral maxillary second molars.^6^

Christie et al^7^ established method in categorizing four‐rooted maxillary second molar abnormalities according to the extent of root separation and deviation. (Table 1).

**TABLE 1:** Classification of four‐rooted maxillary second molar.

Type	Characteristics
I	Two widely divergent palatal roots are often long and tortuous. Buccal roots of tooth are often cow‐horned. Four separate root apices are seen on radiograph
II	Four separate roots are seen, but they are shorter, run parallel have buccal and lingual root morphology and have blunt apices.
III	Constricted in root morphology with the MB, MP, and DP canal engaged in a web of root dentin.

Upon radiological assessment, the case presented in this research demonstrated maxillary second molars having four independent roots of two distinct palatal roots, which are generally lengthy and tortuous, indicating Type I maxillary second molar characteristics.

Inner framework of the maxillary second molar is hard to analyze owing to backward positioning. Owing to the overlapped anatomical components upon this region's imaging, additional palatal root canals cannot be identified. Imaging collected from various angles assist in eliminating superimpositions and enable the professional to identify this unusual aberration.^3^

For acknowledging and handling intricate tooth canal patterns, magnification is presently required.^8^ It contributed to the effective resolution of the current incident.^8^

Cone‐beam computed tomography test can always be used following a thorough clinical evaluation, incorporating traditional radiography.^9^ When a smaller dose of traditional imaging fails to offer enough data during an accurate evaluation, a small FOV CBCT investigation with recreated images in three dimensions may help with diagnosis, treatment plan, and clinical supervision.^9^

From the introduction of CBCT, major advancements in the software and hardware parts have lowered the amount of radiation given to the patient. These enhancements involve modifications to sensor technology, a reduced field of view based on use, and a pulsed radiation approach that adheres to ALARA's radiation dosage principle of “as low as reasonably achievable.”^10^ CBCT dose measurement utilizing multiple CBCT machinery from multiple makers and distinct FOV options, it was discovered that raising the FOV height provides novel and probably radiosensitive tissues to the region of direct contact, whereas raising the width of the beam basically raises the radiation dose to tissues previously exposed to.^10^

Anatomy of upper molars based on CBCT examinations in the Indian population (Table 2).^11^

**TABLE 2:** Anatomy of upper molars based on CBCT examinations in the Indian population.

Number of roots	Tooth	
	First molar	Second molar
1	0	2 (1.4%)
2	2 (1.4%)	12 (8.6%)
3	141 (98.6%)	125 (89.9%)

Abbreviation: CBCT, cone‐beam computed tomography.

Anatomy of upper molars based on CBCT examinations in the Malaysian population (Table 3).^12^

**TABLE 3:** Anatomy of upper molars based on CBCT examinations in the Malaysian population.

Number of roots	Tooth	
	First molar	Second molar
1	0	26 (4.9%)
2	11 (2.3%)	79 (14.7%)
3	469 (97.7%)	429 (80.0%)
4	0	2 (0.4%)

Abbreviation: CBCT, cone‐beam computed tomography.

For dental treatment, CBCT is an important method of diagnosis.^8^ This aided us in distinguishing two distinct palatal canals. According to the information available, maxillary second molar exhibits a greater frequency of anatomical changes than the maxillary first molar, suggesting that its anatomy is considerably more complicated than maxillary first molar.^13^

## 6 CONCLUSION

Comprising the use of CBCT, the present instance illustrates without surgery endodontic treatment on a maxillary second molar containing a pair of different palatal canals and roots, along with two distinct buccal roots and canals. The current study provides a precise comprehension of the canal architecture of maxillary second molar using CBCT scanning. It will assist clinicians understand and foresee the challenges of multidimensional endodontic treatment, particularly when performing chemo‐mechanical preparation. Complicated canal architecture may be effectively identified, controlled, and addressed using a microscope and CBCT.

## AUTHOR CONTRIBUTIONS

**Aishwarya A. Kottur:** Data curation; investigation; writing – original draft. **Abdul Mujeeb:** Conceptualization; data curation; formal analysis; investigation; writing – review and editing. **Niher Tabassum Siddiqua Snigdha:** Writing – original draft; writing – review and editing. **Mohmed Isaqali Karobari:** Conceptualization; writing – original draft; writing – review and editing.

## FUNDING INFORMATION

None.

## CONFLICT OF INTEREST STATEMENT

All authors disclose that there is no actual or potential conflict of interest including any financial, personal, or other relationships with other people or organizations.

## CONSENT

Written informed consent was obtained from the patient to publish this report in accordance with the journal's patient consent policy.

## Data Availability

The (clinical pictures and radiographs) data used to support the findings of this study are included within the article.
